# Prevention strategies to identify LASA errors: building and sustaining a culture of patient safety

**DOI:** 10.1186/s12913-020-4922-3

**Published:** 2020-01-29

**Authors:** Irene Lizano-Díez, Carlos Figueiredo-Escribá, M. Ángeles Piñero-López, Cecilia F. Lastra, Eduardo L. Mariño, Pilar Modamio

**Affiliations:** Clinical Pharmacy and Pharmacotherapy Unit, Department of Pharmacy and Pharmaceutical Technology, and Physical Chemistry, Faculty of Pharmacy and Food Sciences, University of Barcelona, Barcelona, Spain

**Keywords:** Look-alike sound-alike, Medication errors, Drug labeling, Drug safety, Patient safety

## Abstract

**Background:**

Potential look-alike, sound-alike (LASA) errors in outpatient and inpatient prescriptions have been widely described worldwide. However, most strategies of reducing drug name confusion have been only focused on the processes of prescribing and dispensing, often following local rules.

**Main text:**

An illustrative recent example about this topic is given: the antidepressant Brintellix® (vortioxetine) (Takeda Pharmaceuticals USA, Inc.) and the antiplatelet medication Brilinta® (ticagrelor) (AstraZeneca LP). Revision of the initiatives that are currently applied to prevent potential LASA errors in different countries around the world and debate about the emerging strategies that could be implemented in short and mid-term. At present, a common policy worldwide on the authorization of unique names for innovative medicines does not exist. The implication of authorities in topdown strategies and the importance of developing an international health policy on the authorization of unique names for innovative medicines are highlighted in the following piece of opinion.

**Conclusions:**

Building and sustaining a culture of patient safety should be considered as a global top-down strategy which involved all the elements in the system (regulatory bodies, manufacturers and suppliers). The precedent established by the FDA in prevention strategies to identify and avoid LASA errors has been extremely important and should lead to international discussion. Coordinated international efforts are urgently needed in this area for the sake of patients’ safety.

## Background

Since the publication of the reports “To err is human: building a safer health system” [1] by the Institute of Medicine in the US in 1999 and “Building a safer NHS for patients: improving medication safety” [2] by the National Health Service in the UK in 2001, several studies have demonstrated the important role of health professionals in the identification, prevention and reduction of medication errors (ME) and the improvement of safety and quality of patient care [3].

Look-Alike Sound-Alike (LASA) ME caused by the phonetic-orthographic similarity between the names of some medications may have undesirable or adverse effects, especially in chronic polymedicated patients. For instance, the United States Pharmacopeia recorded 26,604 LASA errors between 2003 and 2006 through spontaneous notifications. In particular, 3170 pairs of drug names that could potentially lead to LASA errors were identified; 1.4% of them caused damage to the patient [4]. Similarly, some observational studies suggested that 1% of prescriptions dispensed in the US are associated with LASA errors [5].

Despite these recurrent safety concerns, there is no common policy worldwide regarding the authorization of unique names for innovative medicines. The main reason could be related to the temporary lag that usually exists between marketing authorizations in the US and Europe, which difficult the tracking of brand names for innovations. This timeframe increases dramatically when marketing authorizations are extended to other countries in the world, which also entails higher complexity in terms of linguistics and phonetics. In addition to differences between the US and Europe, there is also high variability between countries of the European Union (EU). In case of centralized marketing authorizations in Europe, there is a working group in the European Medicines Agency (EMA) with representatives of each national agency (27 countries) that ensure that the names of these innovative medicines do not generate conflicts in any countries. However, in case of other authorization procedures different from centralized, the drug agencies of each country check independently the names that are proposed and reject them when they are similar to others already used for marketed products in their own country. This mechanism increases variability in brand names between member states. Even though, paronyms may still exist and homonymous names of medicines from different countries with different compositions could coincide. While there exist strategies for reducing the risk of LASA errors and promoting the current degree of evidence supporting specific ones, the underlying problem still persists. Besides, it is worth mentioning the additional difficulty of idiomatic variability, which could identify LASA errors in different pairs depending on the country of study.

In order to identify which strategies are applied to prevent these potential ME in different countries around the world, we carried out a literature review in PubMed of ME related to LASA medicines applying the MeSH terms (“drug labeling”, “medication errors”, “terminology as topic”, “pattern recognition, visual”, “phonetics”, “recognition (psychology)”) and free terms, reaching a total of 276 results.

## Discussion

### Case report of a medication error by look-alike names

The medicine named Brintellix® (vortioxetine) (Takeda Pharmaceuticals USA, Inc.) represents an illustrative recent example of this problem. Brilinta® (ticagrelor) (AstraZeneca LP) was authorized by the FDA in July 2011 and Brintellix® (vortioxetine) (Takeda Pharmaceuticals USA, Inc.) in September 2013. In the EU, ticagrelor was authorized by the EMA under the name of Brilique® (ticagrelor) in 2010 and Brintellix® (vortioxetine) in December 2013. It should be stressed that Brintellix® (vortioxetine) is subject to additional monitoring since it contains a new active substance authorized in the EU after 1st January 2011, and medicines usually remain under additional monitoring for 5 years.

In July 2015, the FDA issued an alert regarding notifications of LASA errors involving two medicines in both prescribing and dispensing. The drugs concerned were the antidepressant Brintellix® (vortioxetine) and the antiplatelet medication Brilinta® (ticagrelor) [6]. In May 2016, the FDA issued a new safety alert about the name change of Brintellix® (vortioxetine) to Trintellix® (vortioxetine) (Takeda Pharmaceuticals USA, Inc.), with effect from June 2016 [6]. This decision was due to the 50 medication reports describing brand name confusion with (Brintellix® (vortioxetine) and Brilinta® (ticagrelor)) which the FDA had received in just 21 months (between September 2013 and June 2015). In 12 cases out of 50, the wrong medication had been dispensed, though it was not actually administered in any of the cases [6]. In the remaining 38 reports, the error was detected before prescribing and/or dispensing and was classified as a potential error.

The fact that the European authorities did not implement similar measures to those considered by the FDA suggests that either ME of this type appear to have not occurred in the EU, or that none were reported. The first of these assumptions appears rather unlikely, taking into account the rate of events reported in the US and the variety of languages in the EU, which could significantly increase the incidence of LASA errors. Thus, a more likely explanation is that errors were not reported in Europe.

At this point, a serious consideration should be advisable: What is the main reason to justify the lack of reports in the EU about LASA errors? It could be related to the complexity of the system, or to a culture that associates the error with a poor professional performance. With the consolidation of the pharmacovigilance system in EU countries, and since the competent authorities in EU Member States are aware of the adverse reactions associated with ME, the first explanation (the complexity of the system) seems highly unlikely. The second explanation (the association of the error with a poor professional performance) may be more difficult to solve because it is a problem that is culturally ingrained. The study by the American Pharmacist Association entitled “Medication Errors” [7] and the book cited above “To err is human: building a safer health system” [1] both underline the existence of inherent error in human activity, and stress the importance of seeking ways to identify and minimize these errors rather than handing out punishments that do little, if anything, to improve the situation.

### Risk minimization activity

These reports laid the groundwork for patient safety culture and raised the development of highly effective interventions, even out of the hospital setting, such as outpatient care, diagnostic errors, and the use of health information technologies, which are emerging priorities in patient safety. The one-size-fits-all best-practice approaches should be revisited, as most results have been modest and not traceable from place to place due to barriers in implementation and daily practice. Specific tailored strategies should be deployed after identifying the key causes of ME in each setting, organization and country, promoting interdisciplinary cooperation in healthcare setting [8]. The next challenge in patient safety is the development and implementation of tools and top-down strategies that enable organizations to measure and reduce harm both inside and outside the hospital. The National Pharmacy Association (NPA) in UK and the Agency for Healthcare Research and Quality (AHRQ) in the US are currently sponsoring programs in this regard.

Although several countries have implemented strategies for preventing these potential ME, the US has been the pioneer for more than 20 years. Currently, it is the country that has contributed by far most of the evidence on the topic and prevention strategies, in the field of pediatrics as well [9]. The studies published in the UK [10] and some specific projects in Australia, Brazil, Hong Kong, India, Iran, Israel and South Africa are also noteworthy [11–17]. Some examples of confusing names recorded were: cefuroxime-cefotaxime-ceftazidime, doxorubicin-daunorubicin-idarubicin and carbamazepine-oxcarbazepine-carbimazole, as nonproprietary names [11] and Adderall®-Inderal®, PD-Mox®-PD-Rox®, Clomine®-Clozine®, Bioclox®-Biodoxi® and Zyrof®-Zyrop®, as brand names [9, 14].

### What now and what next

Most prevention strategies to identify LASA errors have been developed at hospital setting. Some of the strategies that have been proposed and tested to minimize risks include: 1) computer algorithms that detect potential LASA errors by analyzing names, medication orders and diagnostic claims data [18]; 2) changes in the appearance of labeling and packaging of LASA medicines, paying special attention to their differences (for example the use of uppercase-lowercase letters, boldface or coloured letters to highlight the differences between names; coloured labeling and contrasting background) [19, 20]; 3) inclusion of stickers with security symbols or pictograms on the packaging [19, 20]; 4) re-packaging [17]; 5) bar-code-assisted medication administration [5]; 6) legible manual prescription avoiding abbreviations [3]; 7) avoiding oral and vague prescription [19]; 8) changing order and separate storage of products with similar name, with special attention in case of medicines with narrow therapeutical margin [19]; 9) reminders and alerts for LASA products [19]; 10) correct verification before dispensing and/or administration [5, 19]; 11) the joint use of the brand name and the generic name (in brackets) in prescriptions and drug labeling [19]; 12) review of new medications added in formularies and changes in packaging resulting from contract changes or drug shortages [19].

It is difficult to determine which risk minimisation measures have the most significant impact and there appears to be a lack of good research in this area. More evidence, especially from real-life setting is needed to support safe labeling strategies and the solutions that are not a sustainable solution to the wider LASA challenge. In this sense, Schroeder et al. [21] provide evidence of the association between laboratory-based tests of memory and perception and drug name confusing errors observed in real world, which could be considered as a prevention strategy. By contrast, other authors as Lambert et al. [22] point out some methodological limitations of Tall Man interventions to assess their benefits as effective. Tall Man lettering is an error-prevention strategy used to reduce the risk of look-alike medicine names errors. Tall Man lettering uses a combination of lower and upper case letters to highlight the differences between look-alike medicine names, helping to make them more easily distinguishable.

Many lists have been published in various countries identifying pairs of names that could potentially produce a LASA error. These lists are continuously being updated in the healthcare organizations. As an example, the Spanish delegation for the Institute for Safe Medication Practices has been working with the General Council of Spanish Pharmacists (CGCOF) for more than 10 years to update a database of similar names of medicines and active substances that led themselves to confusion. Currently, this database comprises more than 700 pairs of names [23]. In the field of pharmacy practice, for example, the CGCOF’s health knowledge database (Bot PLUS 2.0), which is commonly used in dispensing, includes alarms regarding the phonetic and orthographic similarity between medicines.

At present, the emerging strategies that could be implemented in short and mid-term are the following: 1) the configuration of computer selection screens and drop down menus in prescription systems to prevent LASA names from appearing adjacent to each other and thus avoid errors; 2) the design of an international system for the validation of names for new drugs/medicines which bears the issue of possible confusions in mind; 3) automated dispensing by means of electronic devices and serialization technology (DataMatrix code), which makes the healthcare supply chain safer as well as more efficient and accurate; 4) use of a closed-loop system with barcode technology to enhance the readability of look-alike labels [20]; 5) consideration of potential LASA errors when ordering stocks; 6) stringent feedback of LASA issues to FDA, EMA and pharmaceutical industry via pharmacists from hospitals and other health professionals; 7) developing strategies to involve patients and their caregivers in reducing risks through information (for example by means of apps that specify the indication for which the medicine has been prescribed and expected appearance, nonproprietary and brand names and potential medication side effects). Artificial intelligence and automated methods will be one of the most accurate tools in the next future to avoid LASA errors and ME in general by minimizing human factors risk.

## Conclusions

Although numerous prevention strategies are being carried out to identify LASA errors, effective top-down strategies for avoiding new LASA names need to be developed. The precedent established by the FDA in this field has been extremely important and should lead to international discussion. Coordinated international efforts are urgently needed in this area for the sake of patients’ safety.

Nevertheless, the implication of all the elements in the system is essential to ensure its success. Most of the strategies focus only on the processes of prescribing and dispensing, but it is vital that regulatory bodies, suppliers and manufacturers should also be fully involved in resolving this serious problem.

## Data Availability

Data sharing is not applicable to this article as no datasets were generated.

## Abbreviations

AHRQ: Agency for Healthcare Research and Quality
CGCOF: General Council of Spanish Pharmacists
EMA: European Medicines Agency
EU: European Union
FDA: Food and Drug Administration
LASA: Look-Alike Sound-Alike
ME: Medication errors
MeSH: Medical Subject Headings
NHS: National Health Service
NPA: National Pharmacy Association
UK: United Kingdom
US, USA: United States of America

## References

[1] Kohn LT, Corrigan JM (2000). Donaldson MS (Institute of Medicine). To err is human: building a safer health system.

[2] National Health System (2001). Building a safer NHS for patients: improving medication safety.

[3] Buurma H, De Smet PAGM, Leufkens HGM, Egberts ACG (2004). Evaluation of the clinical value of pharmacists’ modifications of prescription errors. Br J Clin Pharmacol.

[4] Hicks R, Becker S, Cousins D (2008). Medmarx data report: a report on the relationship of drug names and medication errors in response to the institute of medicines call for action.

[5] Flynn EA, Barker KN, Carnahan BJ (2003). National observational study of prescription dispensing accuracy and safety in 50 pharmacies. J Am Pharm Assoc (Wash).

[6] FDA. Drug Safety Communications. Available at: [https://www.fda.gov/drugs/drug-safety-and-availability/drug-safety-communications] (Accessed 26 Nov 2019).

[7] Cohen MR (2007). Medication Errors.

[8] Bates DW, Singh H (2018). Two decades since to err is human: an assessment of progress and emerging priorities in patient safety. Health Aff (Millwood).

[9] Basco WT, Ebeling M, Hulsey TC, Simpson K (2010). Using pharmacy data to screen for look-alike, sound-alike substitution errors in pediatric prescriptions. Acad Pediatr.

[10] Filik R, Price J, Darker I, Gerrett D, Purdy K, Gale A (2010). The influence of tall man lettering on drug name confusion: a laboratory-based investigation in the UK using younger and older adults and healthcare practitioners. Drug Saf.

[11] Emmerton L, Rizk MF, Bedford G, Lalor D (2015). Systematic derivation of an Australian standard for tall man lettering to distinguish similar drug names. J Eval Clin Pract.

[12] Lopes DM, Néri ED, Madeira Ldos S, Souza Neto PJ, Lélis AR, Souza TR (2012). Analysis of similar drug labeling: potential medication errors. Rev Assoc Med Bras (1992).

[13] Or CK, Chan AH (2014). Effects of text enhancements on the differentiation performance of orthographically similar drug names. Work.

[14] Rataboli PV, Garg A (2005). Confusing brand names: nightmare of medical profession. J Postgrad Med.

[15] Abolhassani N, Akbari Sari A, Rashidian A, Rastegarpanah M (2017). The establishment of the drug naming committee to restrict look-alike medication names in Iran: a qualitative study. Int J Risk Saf Med.

[16] Lavon O, Ben-Zeev A, Bentur Y (2014). Medication errors outside healthcare facilities: a national poison Centre perspective. Basic Clin Pharmacol Toxicol.

[17] Reed AR, Gordon PC (2016). SANS 444:2014: a new standard for small-ampoule labeling and a chance to reduce drug administration errors in South Africa. S Afr Med J.

[18] Figueiredo-Escriba C, Piñero-López MA, Modamio P, Lastra CF, Mariño EL (2019). Medication error due to drug name similarity: an algorithmic approach to orthographic similarity. Lat Am J Pharm.

[19] Ciociano N, Bagnasco L (2014). Look alike/sound alike drugs: a literature review on causes and solutions. Int J Clin Pharm.

[20] Larmené-Beld KHM, Alting EK, Taxis K (2018). A systematic literature review on strategies to avoid look-alike errors of labels. Eur J Clin Pharmacol.

[21] Schroeder SR, Salomon MM, Galanter WL, Schiff GD, Vaida AJ, Gaunt MJ (2017). Cognitive tests predict real-world errors: the relationship between drug name confusion rates in laboratory-based memory and perception tests and corresponding error rates in large pharmacy chains. BMJ Qual Saf.

[22] Lambert BL, Schroeder SR, Galanter WL (2016). Does tall man lettering prevent drug name confusion errors? Incomplete and conflicting evidence suggest need for definitive study. BMJ Qual Saf.

[23] Spanish delegation for the Institute for Safe Medication Practices. [Prevention of errors due to confusion in the names of the medicines]. Available at: [http://www.ismp-espana.org/documentos/view/63] (Accessed 26 Nov 2019).

